# Clinical and Radiographic Evaluation of Success Rate with MTA Plug in Open Apices

**Published:** 2006-04-01

**Authors:** Pari Ghaziani, Akbar Fallah Rastegar, Maryam Bidar, Ghazal Sadeghi, Parviz Chegin

**Affiliations:** 1 *Department of Endodontics, School of Dentistry, Mashhad University of Medical Sciences, Mashhad, Iran*; 2 *Endodontist, Mashhad, Iran*

**Keywords:** Apical Plug, Calcium Hydroxide, MTA, Open Apex

## Abstract

**INTRODUCTION:** The ideal endodontic treatment for the teeth with in complete root-ends and necrotic pulps may involve the use of material which forms an immediate apical barrier instead of long term calcium hydroxide therapy. Such procedure may lead us to a single appointment endodontic treatment. The purpose of the present study was to evaluate clinical and radiographic success rate in necrotic teeth with open apices treated with MTA as an apical plug.

**MATERIALS AND METHODS:** Thirty five patients between the ages of 8-16 with total number of 41 necrotic anterior teeth containing open apices were selected. In the first visit, root canals were debrided and filled with calcium hydroxide. After one week, calcium hydroxide was removed and MTA with the thickness of 3 to 4 mm was put in the apical region of the canals. After 24 hours, the remaining part of the canals was filled with guttapercha and sealer and the teeth were restored with composite. All the cases were evaluated clinically and radiographically after 3 and 6 months and data were analyzed by McNemar test.

**RESULTS:** No clinical symptoms were observed in 97.6% of the teeth after 3 months and in none after 6 months. The study showed a significant difference from pretreatment status. After 3 months radiographic evaluation showed that in 17 cases (41.5%), lesions were reduced and in 14 cases (34.1 %), lesions remained the same, and in 10 cases (24.4%) lesions disappeared. After 6 months in 21 cases (51.2%), lesions were reduced, in 3 cases (7.3%) lesions remained the same and in 17 cases (41.5%) lesions disappeared. These finding were statistically different from pretreatment data.

**CONCLUSION:** The results indicate that MTA can be used as an apical plug in the teeth with open apices following root canal debridement and disinfection with calcium hydroxide. Further investigations with longer follow-ups are recommended in order to evaluate the effect of this material.

## INTRODUCTION

It is difficult to obtain an adequate root canal obturation in the teeth with incomplete root-end formation and necrotic pulps, because of a lack of an apical stop and the presence of thin and fragile walls. Calcium hydroxide has become the material of choice for apexification in order to obtain an adequate apical seal (1).

The high PH of calcium hydroxide may increase alkaline phosphatase activity and the presence of a high calcium concentration may increase the activity of calcium dependent pyrophosphatase (2).

Despite the popularity of calcium hydroxide for apexification, calcium hydroxide therapy has some inherent disadvantages such as variability of treatment time, unpredictability of apical closure, difficulty m patient follow-up and delayed treatment. Therefore, the search is continued for procedures and materials that may allow for apical closure in the teeth with immature apices.

An alternative treatment to long term apexi­ fication procedure is the use of an artificial apical barrier like calcium hydroxide that allows immediate obturation of the canal (3). MTA is a potential apical barrier material with good sealing ability (4) and a high degree of bio­compatibility (5). MTA is a powder consisting of fine hydrophilic particles that sets in the presence of moisture. The major components of MTA are tricalcium silicate, tricalcium alumi­nates, tricalcium oxide and silicate oxide. Hydration of the powder results in a colloidal gel with a PH of 12.5(6). Torabinejad et al. (1993) compared the sealing ability of MTA, amalgam and super EBA as root end filling materials in extracted teeth. The results showed that MTA had significantly less leakage than super EBA or amalgam (4).

Nakata et al. compared the sealing ability of MTA and amalgam in furcal perforations using a dual-chambered anaerobic bacterial leakage model and reported that MTA was superior to amalgam in preventing leakage (7).

Shabahang et al. compared osteogenic protein­1, calcium hydroxide and MTA in root-end induction in dogs and the results showed that MTA induced apical hard tissue formation and was associated with less inflammation than the other test materials (5).

The aim of the present study was to evaluate clinical and radiographic success of endodontic treatment using MTA as an apical plug in teeth with open apices.

## MATERIALS AND METHODS

Patients referred to the endodontic clinic of Mashhad Dental School were evaluated and 35 patients between the ages 8-16 years having 41 necrotic anterior teeth with open apices and signs such as discoloration, swelling, sinus tract, pain in percussion, no response to vitality tests and periapical radiolucency were selected. Clinical and radiographic signs of the teeth were evaluated and the data were recorded. Informed consent was also obtained from all subjects who participated in this research. In each tooth at the first appointment, cavity test was done. If there was no response, the tooth was anesthetized with lidocaine 2% and 1:80000 epinephrine (Darupakhsh, Tehran, Iran). Access preparation was completed and checked for total necrosis. If vitality was observed before reaching the end of the canal, the tooth was omitted from the study Cleaning and shaping the canals were performed using step-back technique. Calcium hydroxide paste (pulpdent, pulpdent corp. USA) was placed in all canals as an intracanal medicament. After one week the calcium hydroxide paste was removed by K-type files (Dentsply, Maillefer, Switzerland) after irrigation with NaOCL 0.5%. The canal was irrigated with normal saline and dried with sterile paper points. ProRoot MTA (Dentsply, Maillefer, Switzerland)) powder was mixed with sterile water in 3:1 Powder to liquid ratio and carried with a carrier to the canal and condensed to the apical end of the root with plugger (NO.1, HU-friedy, Chicago, IL, USA). 3-4 mm apical plug of MTA was created, the extension was checked radiographically, a moist cotton pellet was placed in each canal, and the access cavity was closed with cavit. After 24 hours, the rest of the canal was obturated with gutta-percha (Diadent, Korea) and Roth root canal sealer (Roth international, Chicago, IL, USA). The access cavity was sealed with a bonded composite and a radiograph was taken with parallel technique.

Clinical and radiographic follow-up (3 and 6 months) was performed later.

In clinical evaluation, the absence of swelling, pain in percussion and sinus tract was considered as success.

In radiographic evaluation, two Endodontists evaluated the radiographs according to the following criteria:

1. Increase of radiolucency indicated failure and such cases were labeled as enlargened lesion.

2. No change in radiolucency indicated uncertainty and such cases were labeled as same lesion / unchanged lesion.

3. Decrease in radiolucency indicated some healing and such cases were called decreased / reduced lesion.

4. If the radiolucency almost disappeared, this group was named "complete repair" indicating complete success (8,9).

All data was analyzed by McNemar test.

## RESULTS

**Figure 1:**
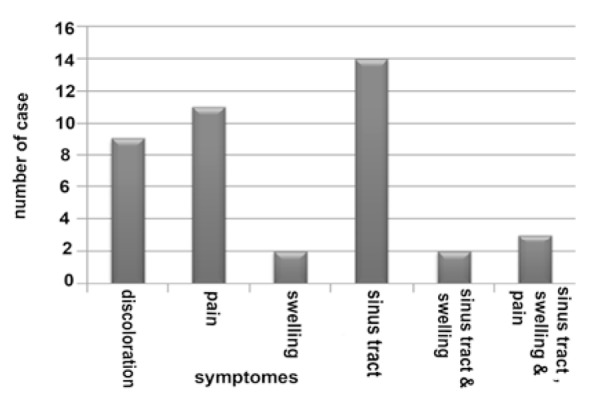
Frequency of pretreatment signs

**Figure 2:**
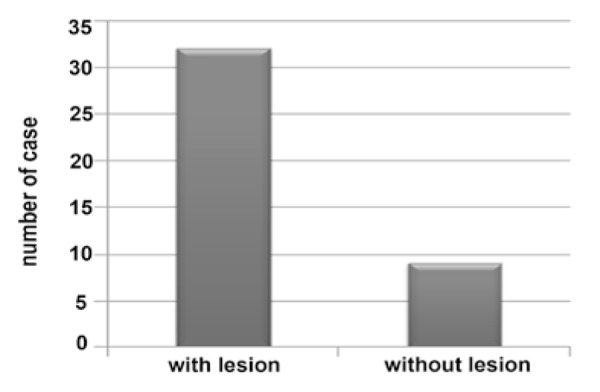
Frequency of pretreatment lesions

Results of pretreatment signs were as follows: 9(22%) discoloration, 11(26.8%) pain in percussion, 2(4.9%) swelling, 14 (34.1%) sinus tract, 2(4.9%) swelling and sinus tract, 3(7.3%) pain, swelling and sinus tract (Figure 1) and pretreatment radiographs showed 9(22%) without lesion and 32(78%) with lesion (Figure 2).

**Figure 3:**
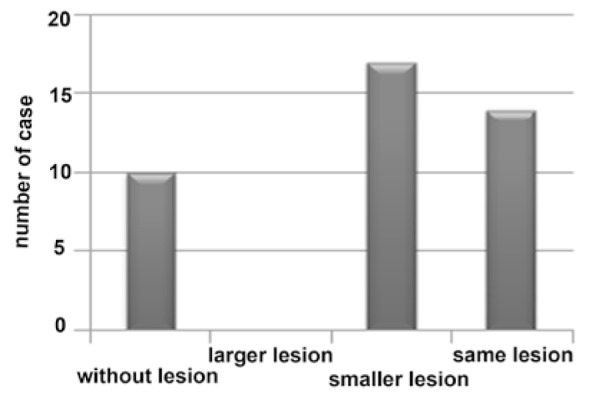
Frequency of post treatment lesions after 3 months

**Figure 4:**
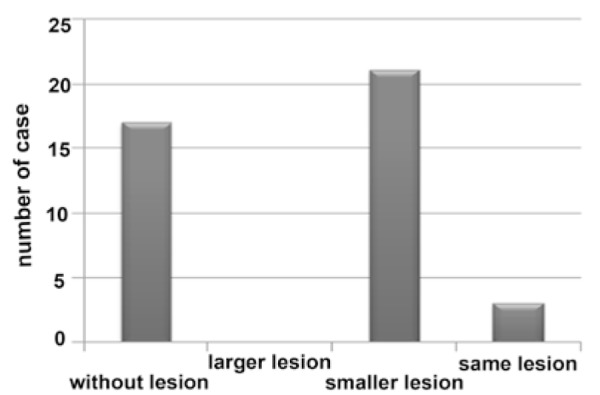
Frequency of post treatment lesions after 6 months

Three month follow ups showed that 97.6% of the samples had no clinical symptoms and this was significantly different from pretreatment status (p<0.01).

Radiographic evaluation after 3 months showed that 10(24.4%) had no lesion, 17(41.5%) lesions had become smaller, 14(34.1 %) had no change, and no larger lesion was detected (Figure 3).

After 6 months, 17(41.5%) samples had no lesions, 21(51.2%) lesions had become smaller, 3(7.3%) lesions had no change and none of the cases developed larger lesions (Figure 4).

At 6-month follow up, 92.7% of the cases showed success radiographically, and none of the patients had clinical signs (p<0.01).

## DISCUSSION

The ideal treatment for open apex teeth involves the use of a material capable of forming an immediate apical barrier. Such treatment is superior to the conventional apexification treatment and can be achieved in a single appointment. Various materials have been used for this purpose including dentine chips, calcium hydroxide, tricalcium phosphate, hydroxyapatite and MTA.

In the present study, MTA was chosen as the material capable of forming such an apical barrier in human teeth because of its reported biocompatibility and superior sealing ability.

Furthermore, MTA has been shown to be a suitable material for one-step obturation in open apices (5).

In our study the method of inserting MTA in the canals was the same as that of Shabahang et al (5). The results of this study were evaluated from two aspects: first, clinical symptoms after 3-months and 6-months follow up, respectively showed 97.6% and 100% success rate and second, radiographic evaluation showed success in 92.7% of cases after 6 months. Since histological studies on human body are not feasible, our study was based on clinical signs and radiographic evaluation.

Coviello and Brilliant in one visit apexification using apical plug with tricalcium phosphate reported that 79% of cases were successful after 9 months (10). Soluti and Sadrin one visit apexification with tricalcium phosphate in cat's teeth reported 91 % radiographic success (11). Fallah Rastegar et al. used hydroxyapatite as an apical plug in cats' necrotic teeth with open apices and reported 94.3% radiographic success (12). In the present study radiographic success after 6 months was 92.7%.

Based on the results of this investigation, placement of an apical barrier such as MTA is suitable alternative to conventional long-term calcium hydroxide therapy. Further investi­ gations are recommended in order to evaluate the effects of MTA in longer periods.
